# Atlanta Academy of Medicine

**Published:** 1875-01

**Authors:** A. W. Calhoun

**Affiliations:** Reporter


### Atlanta Academy of Medicine.

A. W. CALHOUN, M.D., Reporter.

Atlanta, Ga., November 23, 1874.

Dr. Armstrong, President, presiding.

Dr. W. F. Westmoreland reported that the case of suspected extra uterine pregnancy, reported in the meeting of November 17th, was naturally delivered of a small fœtus.

Dr. J. M. Johnson reported another case of phlegmasia alba dolens in the right leg of the same patient whose case was reported and discussed at the last meeting. On last Thursday morning, at 3 o'clock A.M., the same hour as in the other instance, and the same general symptoms, except pain and soreness in the abdomen. There was suppression of the flow, pain in the calf of the leg, extending into the foot, with swelling in the foot and ankle. Patient had continued the use of the liniment, viz: salt, ammonia, camphor, laudanum and oil to the abdomen and left leg; the same was applied to the right, rubbing the limb upward as before, forcing the retained blood and effused lymph and serum upward into the systemic current again. In the course of two hours she was relieved of the swelling and pain; in twenty-four hours the soreness disappeared. After the acute symptoms had subsided, the bandage was applied as in the other leg, and is still on. There is now no pain or swelling. To-day the bandages were removed from the thighs, and the patient is going about quite as much at ease as ladies ordinarily do at this time after delivery.

The Doctor thinks this treatment is new, and is destined to supersede all other methods of dealing with milk leg. In consulting different authors he finds no reference to it. No one he has conversed with has used this particular treatment. The case of Mrs. H., nine years ago, referred to in last week's report, was the first treated in this manner. To this treatment he simply desires to call the attention of the profession, that they may test its availability over other methods.

He has watched this last case closely, visiting the patient often. He is of opinion that, while the disease ostensibly begins in the crural vein, its pathological origin is undoubtedly in the womb. The surface from which the placenta is torn is left in the condition of the stump of an amputated limb, without the advantage of its dressings and washings. Nature has made arrangements for the escape of the foul matters from the festering surface through the os, aided by contractions of the womb. But sometimes there is an irritable os, closing so firmly as not to allow the escape of the disorganized matters within, and before relief can be had the poison is diffused, there is occlusion of the veins, the leg swells by effusions of lymph and serum within the areolar tissue, and phlegmasia alba dolens is upon you.

Upon the authority of Dr. Coe, Dr. W. F. Westmoreland says, that reflex action can account for all the phenomena of milk leg. Where is the data upon which this theory is founded? Dr. Coe maintains that it is not crural phlebitis according to Meigs, and in this he is undoubtedly correct, for it is demonstrable that phlebitis is sometimes the effect and never the cause of milk leg; but falls into an error equally great in ascribing it to reflex action because there is pain in the lumbar and sacral regions, and also in the vulva and perineum. It is much easier to account for this upon other hypotheses, as he would try to show. The hypogastric artery is given off from the external iliac at the last lumbar vertebra, and ramifies the womb through the connective tissues, (utero peritoneal substance) while the hypogastric veins return the blood from the womb, the nates and muscles of the sacrum. The pudic artery also ramifies the uterus, and the blood in returning enters the internal iliac through the pudendal veins; then there are the right and left ovarian and two uterine arteries, with returning veins, called the uterine plexus, the latter conveying their blood also to the hypogastric. Thus it will be seen that an organ so vascular as the womb, with its arteries and veins and a vast network of capilaries also, the office of which is not only the nutrition of the part, but the carrying back of all the blood thus used into the systemic circulation—having ramified the womb in every part, and been brought into contact with the denuded surface from which the placenta has been removed, would, on its return, carry with it disorganised and poisonous matters, driven outwardly through the sinuses by the contractions of the womb upon a mass of fluid poison, retained there by an occluded os. The blood in the womb, although poisonous, is nevertheless homogeneous, and might not interrupt the functions of the veins, conveying it to the iliac; but upon its contact with healthy blood, plugs up the vein at once, and produces the phenomena he was trying to account for.

From this reasoning, cannot all that is claimed for Dr. Coe's theory be explained? We all admit the wonderful power of the nervous system in complicating pathology, diagnosis and treatment. It is almost a sealed book up to this time. Our business is to keep within the pale of legitimate deduction, and not venture where the light of revelation has not penetrated. Virchow introduced the terms thrombi and emboli in describing this and other diseases in 1847. These two words—simple words if you choose to call them so—are nevertheless potent words, for they point to a disease and a pathology which had never been touched upon except by Robert Lee in 1844.

One hundred years ago it was known that vegetations formed about the heart and other places, and that death resulted from it; but it remained for this great author and pathologist to show the basis of the morbid changes with their consequences. Pus produces embolism; may not other poisons do so too? It is asserted and taught that, after death takes place, decomposition begins in the nitrogenous compounds. In a word, the very element that organises and builds is the same that disorganises and pulls down. A process of fermentation takes place then which is imparted to the other elements; and as they began by cells and molecules to build, so by cells and molecules they pull down. May there not be a fermentative property in ichorous matters like to the one just alluded to, capable of producing thrombus in the crura? He admits the difficulties in the way of getting rid of a pus thrombus—the difficulty of removing it and the consequences following the breaking into emboli. In none of the cases he had treated in this way had there been any difficulty or evil consequences from removal of the thrombus by forcing the blood through the vein, destroying the plug and restoring the blood to the general circulation, and with it all the serum and lymph contained in the intervening areolar substance.

The Doctor thinks his plan entirely new, and does not doubt its invariable success when commenced early. His other treatment consists of black drop enemas as often as pain is felt. For this purpose 30 drops in two teaspoonsful of warm water should be thrown into the rectum whenever necessary to procure quietor relieve pain. The injection should be given with an ounce or half ounce syringe, with a long tube, say one inch, so as to make it certain that the fluid enters the bowel beyond the sphincters. He also gave carb. ammonia in five grain portions, every three hours, to assist in keeping the blood in a fluid state, and used over the abdomen and to the limbs, during the rubbing process, a liniment of salt, aqua ammonia, camphor spirits, laudanum and sweet oil. He used a douche of hot water to the womb every hour or two, to restore the suspended lochia, and to keep it up afterwards. He has treated dysmenorrhœa for many years with hot water, and used it similarly in this case.

Dr. W. F. Westmoreland said that Dr. Johnson's treatment was new, and certainly demanded the consideration of the Academy. In the two cases reported, Dr. J. had, in a few hours in one and a few days in the other, aborted or cut short a disease which, in the majority of cases, is several weeks running its course. Black drop per rectum and warm applications to the womb, in the form of vaginal enemas of tepid water, long continued, would tend to remove irritation of the womb, as such applications do in other tissues. He differs with all who believe that phlegmasia dolens is a disease of these veins; and particularly did he differ with those who contend that this disease is an inflammation of the crural vein. Regards the inflammation of the veins observed in post mortem examinations as secondary, or an accident, occurring during the progress of the disease, and not the essential lesion in phlegmasia dolens. The symptoms and the results of treatment in the case reported by Dr. Johnson to-night, as well as the case reported a week or two ago, strengthens his belief in the correctness of Dr. Coe's theory, as presented by him at the last meeting of the Academy. Evidently, in both cases, the disease was ushered in by a train of nervous symptoms. The pain in both cases, particularly the pain in the calf of the leg, can only be accounted for by the theory of reflex action. Does not think that a disease of the veins, or the theory of embolism, will account for the train of symptoms usually present in this affection. The veins which convey the blood from the womb are the uterine and vaginal plexus, which, in part, makes the internal pudic vein; this empties into the internal iliac vein, which, with the external iliac, make the primitive iliac. All the blood going to the womb is returned through the pudic vein, unless we have a small portion returned through the ovarian vein, which empties into the inferior cava on one side, and the renal vein on the other. If, as some suppose, the contents of the womb in part enters the veins which are patulous after parturition, it is likely to affect the vessels with which it first comes in contact, producing, in the opinion of some, phlebitis. He asks, how can we account for the symptoms in the two cases reported by Dr. Johnson, by a disease of the uterine plexus, the pudic, or even the internal iliac veins? How can a disease of any character, of either one or all the vessels mentioned, produce a pain in the calf of the leg, or the swelling of the limb, both of which appear early in this disease, and, in the cases under discussion, were the first and most prominent symptoms? He does not think that the swelling of the limb can be accounted for upon the theory of a mechanical obstruction of the veins, as it has already been shown that the veins most likely to be diseased have nothing to do with conveying the blood from the swollen limb. He is of the opinion that if the fluid contained in the womb, whether it be in the form of pus, decomposed animal matter, or other noxious fluid, enters the veins producing coagula or emboli, as some have supposed, this effect upon the circulating fluid would be produced where such impure matter came in contact with the blood, and thus obstruct the circulation, if obstructed at all, at the point where the coagula would most likely form, and not the circulation in the external iliac or femoral veins. He does not think it reasonable to suppose that the coagula are forced through the swollen veins, to be arrested in the larger, the primitive iliac, for example, and thus arrest the circulation in this important vessel. Neither does he think it reasonable to suppose that this mucous fluid should be carried through the uterine plexus, internal pudic, and internal iliac veins, to form a coagulum in the primitive iliac. But he contends that, even admitting this improbable result, it is still not sufficient to account for the phenomena in phlegmasia dolens.

Dr. Miller investigated this subject years ago, and has not found cause to change his opinion. The disease had existed one hundred and fifty years before dissections were made. Since that time these have shown uniform appearances. Examination shows some of the veins blocked up, and sometimes an organized plug; sometimes pus exists. He cannot see how nervous irritation can account for the appearances. Dr. Robert Lee believed the origin of the disease to be in the hypogastric veins, and that the trouble in the crural vein was secondary. The internal iliac is most marked in the disease. In many respects it differs from phlebitis. Phlebitis is fatal; phlegmasia dolens is not. The phenomena are different in the two. Henry Lee introduced pus into the jugular vein of a horse, and instantly the blood in this entire vein was coagulated. It is known that the veins in the iliac region are pluged up, beginning always in the internal iliac. How does it occur without inflammation? He is of the opinion that it is produced by substances from the uterus. When a vein is wounded it contracts, and prevents the entrance of pus, but if the wound be in the bone it can get into the current of circulation. Hence multiple abscesses are found more in wounds of the bones. When the womb increases in size the vessels dilate, and the discharge from the uterus enters into the veins. He thinks pus enters from the uterus, or some other discharge producing a like result. You have the sudden invasion of the disease, the obstruction of the circulation, which may occur at many points, pain, etc., beginning in the limb. There is also tumefaction of the vulva, nates, etc. The blood becomes coagulated by the entrance of pus, and the nervous trouble depends upon the obstruction of the blood. Swelling will remain until the circulation is established in other directions; this accounts for the slow and imperfect recovery. Blocking up of the vessels is essentially the disease. Some of the rare terminations of the affection are, gangrene, suppuration at various points, death upon sudden exertion; the coagulum being detached and passing into the heart. If the secretions in the womb produce the disease, then cleanliness of the parts must be kept up. Friction would do no good after the coagulum has become firm. All puerperal diseases are attributable to like causes, the circulation being deteriorated by the entrance of foul matter from the womb.

Dr. Woodhull—If the views of Dr. Miller are correct, would not the loosening of the clot by friction be dangerous?

Dr. J. M. Johnson had the opportunity of testing the treatment at the very beginning of the disease. He had seen milk leg bandaged when a boy. What was the object of this but to clear off the accumulated fluids? If rubbing out the fluids will relieve the trouble, he does not think there is much danger of a post-mortem.

Dr. J. G. Westmoreland introduced a case of Dr. Dickey's, of partial paralysis, and asks if any plan can be suggested as to treatment. Referred to a special committee. Adjourned.

ATLANTA, GA., December 1, 1874.

Dr. Armstrong, President, presiding.

Dr. W. F. Westmoreland showed an instrument, devised by himself, for applying medicines to the interior of the uterus, (for instance, pulv. nit. silver and nit. potass, etc.)

Dr. Taliaferro has under treatment an unmarried lady, 18 or 19 years of age, who, five or six months ago, became troubled with a kind of nervous spell, which he supposed to be hysteria. Her health was broken down and her menstruation was sparse. She was of a melancholy disposition, and kept herself aloof from society. Previous to menstruation, with which began the spells, she was in perfect health. Latterly she has the attacks between the menstrual periods, and loses consciousness for a moment or two. He thinks there is a sort of blending of hysteria and catalepsy. An examination showed congestion about the cervix and vaginitis. There was also retroflexion and tenderness of the organ. Upon restoring the uterus to its proper position, the inflammation and tenderness disappeared. Her general health is now restored, and she enjoys herself, but she still has these attacks, and scanty menstruation, the show lasting usually for two days. When she wore the galvanic stem she menstruated freely and normally; but, living out of the city, she had to cease the use of the stem. Even while menstruating normally under the use of the stem, she would still have an attack upon the approach of menstruation. In the last few weeks she had had a convulsion, which was relieved to some extent by the use of bromide potassium; she is now taking bromide lithium. There was no pain during the menstruation. He had supposed there was a want of development in the ovaries. He asked to hear the Academy as to the pathology and treatment of the case.

Dr. W. F. Westmoreland thought it was often the case that these troubles did not depend upon the condition of the womb. Here we have all the womb troubles corrected, and still the disease continues to exist. We frequently attribute nervous affections to the womb, when this is not the cause, and hence treatment does not remove them. A lady patient of his had similar symptoms to Dr. T.'s case, and examination showed nothing remarkable. A few applications relieved the tangible symptoms, and a tonic treatment was also of some benefit. But in these cases self-abuse is not unfrequently a cause, as in his own case; for the patient ultimately acknowledged the fact.

Dr. Cumming said that in Europe they develop the ovaries by treating the mammary glands. The connection between the two is so intimate that treating the latter brings about a good result in the former. This is too much the case with boarding-school girls—the girl does not develop into a woman. If the case mentioned was in a school girl, he would recommend fresh air, horse-back exercise, electricity, etc.

Dr. J. M. Johnson is of the same opinion as Dr. Taliaferro. We may have partial menstruation, and yet complete ovulation. He has in charge the case of a married lady, who, though much reduced, and passing only 1 to 1½ teaspoonful at each period, became pregnant, and is now much better. Many of these troubles are cured by marriage. If this individual were to marry and become pregnant, she might be cured. Masturbation is sometimes practiced by young females; it is hurtful and productive of catalepsy and hysteria. Matrimony is the remedy he would recommend—take away the inclination to abuse, and recommend marriage. Out-door exercise and employing the mind is most excellent. Take her mind from novels, and let her work and tire herself enough to make her sleep, and she would get well.

Dr. Battey continued the report of a case discussed before the Academy last spring, where it was thought that the spleen was dislocated. He had an opportunity of examining the case again to-day, and is confirmed in his diagnosis. The tumor presents clearly the outlines of the spleen. During the summer she has been taking muriate ammonia, with the view of reducing the tumor. Her health is much improved, and she thinks the tumor has diminished.

Adjourned.

---

ATLANTA, GA., December 8, 1874.

Dr. Armstrong, President, presiding.

Dr. Battey exhibited a boy of seven years, upon whom he had operated for *hare-lip* four days ago, by a method, as regards the approximation and securing of the pared surfaces during the process of union, peculiar to himself, which will be found illustrated and explained at page 198 of the American reprint *London Lancet,* for the year 1860. The peculiarity of his method consists in the application of a combined splint and compress over the line of proposed union, held in position by silver wires passing deeply through the lip, down to the mucous lining, and outwards through perforations in the splint, upon which they are secured by compressed shot. This arrangement allows of a straight and accurate coaptation of the pared edges, which are drawn firmly up to, and very smoothly moulded by, the leaden splint and compress; by which means the puckering action of the old process by pins and twisted sutures is wholly avoided, and a straight, uniform and very narrow line of cicatrix secured, which is much less observable than that which results from the hare-lip pins.

The splint is useful likewise in stretching the lip downwards, and serving, to a great extent, to prevent the pro-labial notch which so often remains. It likewise covers up the wound, excludes the air, and protects the parts against external sources of injury. The splint, in the case presented, has been removed to-day. In consequence of the broadly rounded corners of the fissure in this case, it was found impracticable to pare the edges so deeply as to cut out entirely the pro-labial notch without sacrificing entirely too much tissue, and rendering the upper lip too narrow. He therefore executed the paring after the manner of Malgaigne, so far as to leave a little flap on either side, which flaps are brought down and united to fill the notch. If, in the case presented, this shall prove to be insufficient, it is proposed, at a future time, to make a slight horizontal incission just above the cutaneous border, by thrusting a narrow bistoury entirely through the lip, and then approximating, by a single silver wire, the two extremities of the incission in such manner as to change the direction of the incised line from horizontal to perpendicular, as illustrated to the Academy upon the bit of soft buckskin. By this means the cicatricial line will be increased in length to nearly the extent of the little incission made in the lip, and *per consequence*, the labial border let down at that point, and the notch effaced. This device, Dr. B. states, is likewise original with himself, but whether he is the first to enunciate it to the profession or not he is unable to say.

Dr. W. F. Westmoreland demonstrated several plans usually practiced for the relief of hare-lip, by which the pro-labial notch could be prevented. He thought Dr. Battey right in dissecting up the lip from the sub-maxilary bone, for this is absolutely essential to a good cure.

Dr. DeWitt reported a case of powder burn in the face, where, after washing away much of the powder with tepid water, he used a light-bread poultice, which was continually kept moist.

With the exception of four spots, the face and eyes are entirely clear of the powder.

Dr. Battey was consulted by a friend with an abrasion upon the side of the face, caused by a firebrand, in which there was some powdered charcoal. It did not come away with the scab, as expected, and he mentions the case to show that such things should be at once removed.

Dr. Boring reports that his case of supposed gout was still suffering, and nothing seemed to do any good. He has tried electricity and everything else; local applications have no effect. Chloral and bromide potassium is all that gives relief. He tried Dr. J. G. Westmoreland's suggestion of a blister to the spine, but it did no good whatever.

Dr. Woodhull asked if the Academy had had any experience in the use of black snake root (cimicifuga) in the treatment of muscular rheumatism. He had tried it in some cases with good effect.

Dr. Battey had a case where, after labor, the symptoms were similar to those in Dr. Boring's case. He thought it to be neurosis, and connected the pain in the feet with pressure upon the anterior sacral nerves; the pain was mostly in the sole of the foot.

Dr. DeWitt had scarcely ever failed with prickly ash and snake root (cimicifuga) in chronic rheumatism.

Dr. W. F. Westmoreland reported two cases of stricture, the result of gonorrhœa—to show the rapid and tardy cure—one at the bulbous portion of the urethra, the other 1½ inch further in front. The filiform bougie was used with difficulty. Sir H. Thompson's divulsor broke it up without any constitutional symptoms. Immediately afterwards a No. 16 or 17 steel bougie was introduced, and allowed to remain one to one and a half hours. In seventy-two hours the patient was up and about. The second case was of longer duration, the result of stone in the urethra. There was complete retention of urine for three days. A filiform bougie was introduced easily, and the perineal section was made. The bladder was opened, and he is now urinating through the opening. He is going about everywhere. He can now operate upon the stricture and make the operation complete. It is best to wait until the constitutional effects of the perineal section have disappeared before operating upon the stricture; for otherwise it is dangerous.

Adjourned.

